# Preparation of Hydrophobic Cryogel Containing Hydroxyoxime Extractant and Its Extraction Properties of Cu(Ⅱ)

**DOI:** 10.3390/gels10010009

**Published:** 2023-12-21

**Authors:** Hayato Takase, Naoto Goya, Shiro Kiyoyama, Koichiro Shiomori, Hideki Matsune

**Affiliations:** 1 Department of Chemical Engineering, Graduate School of Science and Engineering, Kagoshima University, 1-21-40 Korimoto, Kagoshima 890-0065, Japan; takase.hayato@cen.kagoshima-u.ac.jp; 2 Department of Applied Chemistry, Graduate School of Engineering, University of Miyazaki, 1-1 Gakuenkibanadai-nishi, Miyazaki 899-2192, Japan; 3 Department of Chemical Science and Engineering, National Institute of Technology, Miyakonojo College, 473-1 Yoshi-cho, Miyakonojo-shi, Miyazaki 885-8567, Japan; shiroh@miyakonojo-nct.ac.jp; 4 Department of Applied Chemistry, University of Miyazaki, 1-1 Gakuenkibanadai-nishi, Miyazaki 899-2192, Japan; shiomori@cc.miyazaki-u.ac.jp

**Keywords:** hydrophobic cryogel, cryo-polymerization, frozen temperature of organic solvent, supermacroporous structure, extraction properties of Cu(Ⅱ)

## Abstract

Hydrophobic cryogels with monolithic supermacropores based on poly-trimethylolpropane trimethacrylate (pTrim) containing 1-(2-Hydroxyl-5-nonyphenyl)ethanone oxime (LIX84-I) were successfully prepared by a cryo-polymerization technique using organic solvents with freezing points between room temperature and around 0 °C as solvents. The prepared cryogels were characterized in terms of macroscopic shape and porous structure. The cryogels had a monolithic supermacroporous structure and high contents of LIX84-I depending on the added amount of the extractant to the monomer solution. The amount of LIX84-I impregnated in the cryogel had a linear relationship with the added amount of LIX84-I in the monomer solution for cryo-polymerization. Cu(II) in the aqueous solution was immediately adsorbed into the cryogel containing LIX84-I.

## 1. Introduction

Cryogels are porous materials formed by polymerization (cryo-polymerization) of polymer precursors, which are solutions containing monomers, crosslinking agents, and polymerization initiators, and occur at the freezing point of the solvent of the solution [1,2,3]. During the process of cryo-polymerization, a phase separation occurs between the unfrozen region where the polymer precursor is present (concentrated monomer) and the frozen region of the solvent (ice crystals) [4,5,6,7]. Polymerization occurs only in the unfrozen region of the system, and the frozen crystals of the solvent act as a porogen [8,9,10]. After polymerization, the solvent crystals can be thawed resulting in the formation of a porous structure in the gel on the micro-meter scale, which is known as the “supermacroporous structure” [11,12,13]. The nature and type of solvent and monomer or polymer precursor, the temperature of the cryopolymerization, the rate of freeze–thaw dynamics, and several other factors affect the properties of cryogels [14,15,16]. Generally, water is used as the solvent, and the polymer composition, such as hydrophilic or hydrophobic monomers, and the preparation method used depends on the application. Cryogels with hydrophilic polymers are used for selective recovery or removal of useful or toxic substances from the aqueous phase by introducing functional groups into the polymer. Cryogels complexed with solid particles such as activated carbon [17,18,19,20], inorganic particles [21,22,23], and porous polymer particles [24,25] have also been reported. Various techniques are employed for the preparation of cryogels [26,27]. For practical applications, the materials are designed by modifications such as coating [28,29], hybrid [30,31], impregnation [32,33,34]. Thus, the application of cryogel can be expanded across wide fields; for example, in separation [35], pharmaceuticals [36], healing of wounds [37,38,39], sensors [40,41,42], and the cosmetic industry [43]. The supermacroporous structure of the cryogel allows water to move easily from the outside to the inside of the gel, which results in rapid transfer of the solute to the microparticles embedded in the cryogel wall and rapid adsorption by the internal microparticles compared to conventional hydrogels [44]. Adsorption separation of various substances has been achieved by selecting the microparticles. Hydrophobic cryogels, which prefer oil to water, have been reported to be useful for the adsorption and separation of organic substances from aqueous solutions [7,45].

We have been studying extraction microcapsules, which are constructed from a spherical body containing interconnected spherical pore-containing extractants in the wall part of the spherical and porous particles [46] and extractant-impregnated porous particles [47]. An extractant is a specialized functional molecule that selectively interacts with and extracts metal ions in the solvent extraction method from an aqueous solution. The porous microcapsules and porous polymer particles interconnect with spherical pores on the surface and their inside, which are prepared using W/O/W emulsions as a template [7,46,47,48]. It has been found that by using microcapsules or microparticles with interconnected spherical pores, the aqueous phase moves quickly into the microparticles, and the extraction rate of metals is very fast [49]. Cryogel, which has a structure similar to the structure of these linked spherical pores, is expected to have an excellent structure as a carrier material for extractants. Since extractants are generally dissolved in organic solvents in general solvent extraction, extractants are often hydrophobic [50,51,52]. When the extractant is encapsulated in cross-linked hydrogels, the hydrophobic extractant is encapsulated as dispersed small droplets in the hydrogels. Although the hydrogel microcapsules were easy to prepare and the encapsulation efficiency of the extractant was high, the extraction rate was moderately slow, and the extraction rate remained almost the same when the size of the dispersed droplet was changed, although the particle size was almost the same and the amount of encapsulation was the same. This suggested that the diffusion of metal ions inside the gel dominated the extraction rate [53]. On the other hand, the microcapsules and the microparticles have a hydrophobic wall, and the extractant is encapsulated in the wall. Hydrophobic cryogels are also considered to be suitable for encapsulating extractants in the wall of the cryogel. Therefore, in this study, a porous polymer material was prepared by dissolving the extractant in a solution of organic solvent and hydrophobic monomer and preparing it under cryogel conditions to encapsulate the extractant in the porous wall created by the hydrophobic polymer. The extraction of copper ions was performed using this porous material, and the extraction properties were clarified.

## 2. Results and Discussion

### 2.1. Preparation of Hydrophobic Cryogel

**Figure 1:**
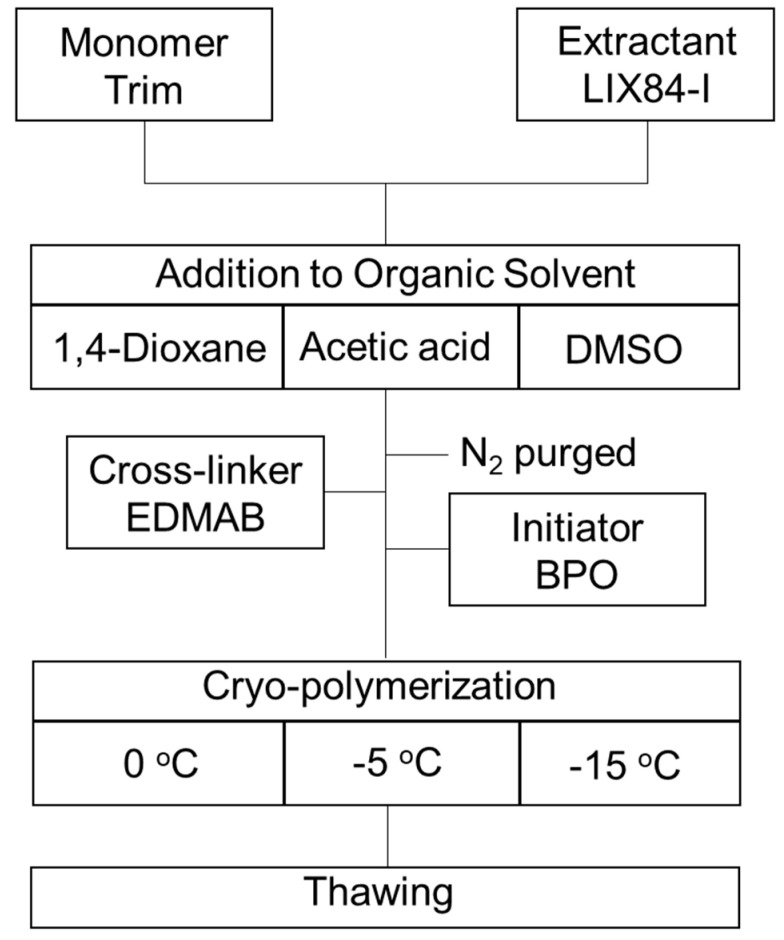
Preparation scheme for hydrophobic cryogel containing extractant LIX84-I.

Cryogel is a polymer material that can be prepared by polymerization at the freezing temperature of the solvent used. The hydrophobic cryogels were prepared using trimethylolpropane trimethacrylate (Trim) as a monomer with cylindrical shape. Trim forms a hydrophobic cryogels [7] and can be expected to impregnate the hydrophobic extractant in the polymer network of the cryogels. The cryogels were prepared at various conditions to construct the preparation condition for the impregnation of a hydrophobic extractant, 1-(2-hydroxy-5-nonylphenyl) ethanone oxime (LIX84-I), which is used for the extraction of Cu(II) from aqueous solution in solvent extraction system [54] in the polymer network constructing the cryogel wall. The hydrophobic cryogels containing LIX84-I were prepared following Figure 1.

#### 2.1.1. Macroscopic Structure of Hydrophobic Cryogels Prepared with Various Solvents

**Figure 2:**
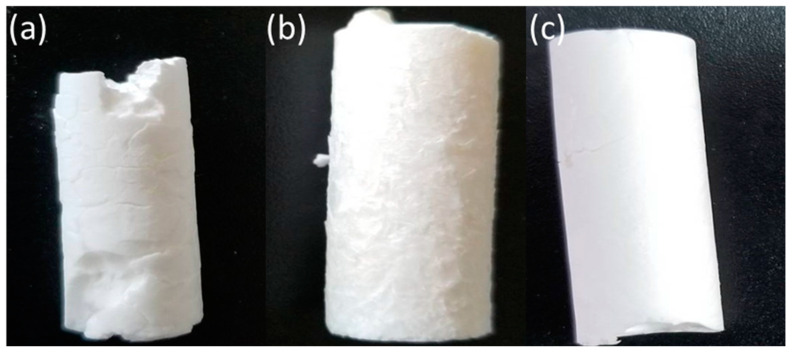
Photograph of hydrophobic cryogels. The hydrophobic cryogels were prepared at −15 °C with using organic solvent (**a**) DO, (**b**) AcOH, and (**c**) DMSO.

The texture of cryogels (i.e., appearance, porous structure, mechanical strength) would be affected by the solvents, such as dioxane (DO, m.p. 11.8 °C), acetic acid (AcOH, m.p. 16.7 °C), and dimethyl sulfoxide (DMSO, m.p. 18–20 °C), which are used for the preparation of cryogel. Herein, the macroscopic structure of hydrophobic cryogels prepared at −15 °C using three organic solvents was observed, as shown in Figure 2. All cryogels prepared with various organic solvents were prepared with cylindrical shape. Under various organic solvents, it was clear that the monomer (Trim) caused the polymerization in the unfrozen region at frozen temperature of the solvent. However, compared to hydrophilic cryogels prepared with water as solvent, less flexible and relatively hard polymeric materials were obtained in the case of all organic solvents. Furthermore, in the case of DO, brittle and fragile polymeric materials were obtained rather than those of AcOH and DMSO. AcOH and DMSO were found to be more suitable solvents to be used than DO under these preparation conditions.

Since the polymer precursor was transferred into a syringe vessel and cryo-polymerized during the preparation process, the macroscopic shape of the prepared cryogels will be the same as that of the vessel. By selecting the type of the vessel, the macroscopic shape of cryogel can be designed to be cylindrical, sheet-like, and star-shaped, etc. [11]. Hence, the cryogels prepared here are expected to be deployed in devices and other engineering applications due to the ease of controlling the macroscopic shape and adjusting it to the appropriate shape. Further study would be needed to overcome the brittle and fragile polymeric materials in the case of DO.

#### 2.1.2. Observation of Porous Structure of the Hydrophobic Cryogels

**Figure 3:**
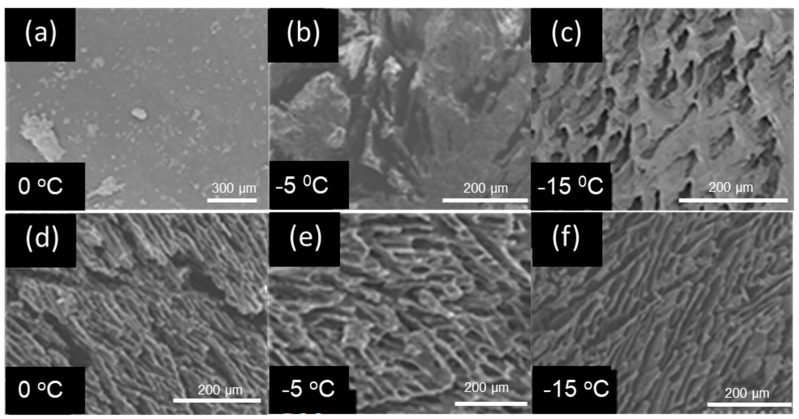
Effect of type of organic solvent and polymerization temperature on the morphologies of the hydrophobic cryogels prepared in AcOH and at (**a**) 0 °C, (**b**) −5 °C, and (**c**) −15 °C; and in DMSO at (**d**) 0 °C, (**e**) −5 °C, and (**f**) −15 °C.

Cryogel have a unique porous structure which is hierarchical and has interconnected large pores (10–200 μm) [11]. Thus, these porous structures are called as “supermacropore” because they are significantly larger than IUPAC-definition macropore (>50 nm) [11]. Since phase separation is caused between the frozen region and the unfrozen one during cryo-polymerization, the supermacroporous structure is formed [11]. Here, the solvent crystals and concentrated polymer are adopted as phases: the frozen region and non-frozen one. Hydrophobic cryogels were prepared in various conditions, such as (i) using AcOH or DMSO as an organic solvent, and (ii) cryo-polymerization temperatures at 0 °C, −5 °C, and −15 °C. The results were observed by using Scanning Electron Microscopy (SEM), as shown in Figure 3. When AcOH was used as an organic solvent at −5 °C and −15 °C, the formation of continuous large pores and layered walls inside of the polymerized materials were formed, confirming a supermacroporous structure. However, when AcOH was used to prepare the cryogels at 0 °C, no pores and no layered structures were formed inside of the formed polymeric solids, suggesting no phase separation (Figure 3a). Although the solidification of the solution was observed at 0 °C, however, the solidified materials of the reaction solution formed homogeneous solid and no clear phase separation between the crystalline and non-frozen phases of the solution.

When DMSO was used for the cryopolymerization, a continuous and inter-connected large pores and layered wall structure was formed inside of the polymerized materials as shown in the SEM images in Figure 3d–f. All hydrophobic cryogels were achieved in preparation conditions for cryo-polymerization temperatures in the range of 0 to −15 °C with using DMSO.

#### 2.1.3. Porous Properties of Hydrophobic Cryogels

**Figure 4:**
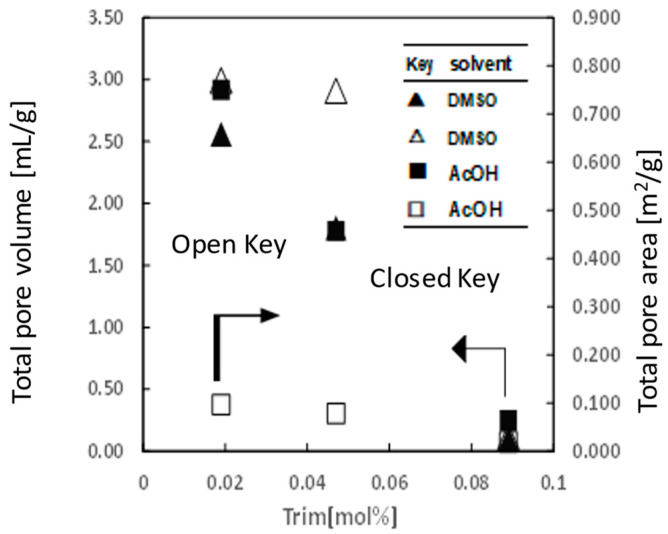
Effect of molar concentration of Trim in the reaction solution on the porous properties of the hydrophobic cryogels prepared with AcOH or DMSO and at −15 °C.

The porous properties of cryogel were changed by the preparation condition and evaluated by using mercury intrusion porosimeter (MIP) analysis. Here, the porous properties of cryogels were characterized in relation to the kind of organic solvent used for the polymerization, the cryo-polymerization temperature, and the concentration of monomer, Trim, used in the preparation. Table 1 systematically compiles the porous characteristics of cryogels produced under various conditions, encompassing total intrusion volume, total pore area, and *D_v_*. It was found that the porosity of cryogel was gradually decreased with increasing temperature of cryo-polymerization in AcOH and DMSO. In addition, the relationship between the amount of monomer, Trim, used for the preparation of cryogel and their porosities was investigated (Figure 4). The hydrophobic cryogel was prepared with AcOH or DMSO at −15 °C. The total pore volumes of the cryogels prepared with DMSO and AcOH had almost same values and decreased linearly with an increase in the molar concentration of Trim in the preparation solution. However, the total pore area of the porous cryogel prepared with DMSO was larger than that of AcOH and almost constant at the lower concentration of Trim and decreased at high concentration of Trim used for the preparation of cryogel. These differences between total volume and pore area on the hydrophobic cryogels with DMSO and AcOH have been unclear. More investigation of the formation mechanism and effect of solvent type on the phase separation and porous structure formation during the cryopolymerization would be needed in the future work.

#### 2.1.4. Content of LIX84-I in Hydrophobic Cryogels

**Figure 5:**
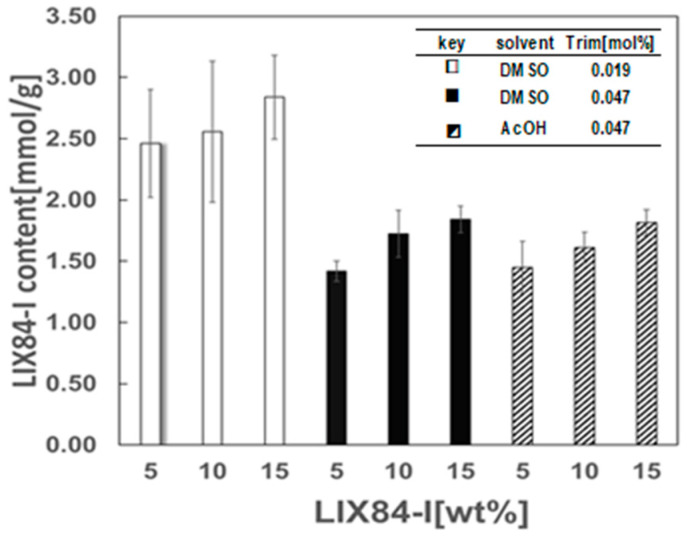
Effect of concentration of LIX84-I in the reaction solution on the LIX84-I contents in the cryogels prepared with DMSO or AcOH, at 1.9 × 10^–2^ and 4.7 × 10^−2^ mol% of Trim, and at −15 °C.

To expand the application of cryogel as separation materials, the hydrophobic cryogel carrier was modified with extractant LIX84-I and functionalized. The hydrophobic cryogel containing LIX84-I were prepared by mixing LIX84-I in the polymerization precursor at the preparation step. The concentration of LIX84-I in the polymerization precursor prepared with AcOH or DMSO at −15 °C was changed and the content of LIX84-I in the cryogels was measured as shown in Figure 5. The LIX84-I content in the cryogels increased with an increase in LIX84-I concentration in the polymerization precursor in the both cases of DMSO and AcOH. When DMSO was used as solvent and the Trim concentration was low, LIX84-I content was higher than that of high Trim concentration. This means LIX84-I amount in the wall of cryogel at low Trim concentration become larger than that of high Trim concentration. In both cases of DMSO and AcOH at the same Trim concentration, LIX84-I contents in the cryogels were kept at same LIX84-I concentration in the precursor solution. This means the LIX84-I amount in the cryogels would be the same as the monomer amount compared with the LIX84-I amount in the cryogels. The addition of LIX84-I in the precursor solution showed no change in pore structure and layered wall structure with those without LIX84-I. Therefore, the hydrophobic porous cryogel containing LIX84-I is expected to be used as a separation material for various metals by utilizing the function of LIX84-I, especially Cu(II) extraction from aqueous solution.

### 2.2. Extraction Properties of Cu(II) by Cryogel Containing LIX84-I

The hydrophobic cryogel containing LIX84-I was applied for the batch extraction of Cu(II) in the aqueous solution. The cryogels containing LIX84-I was added to the Cu(II) aqueous solution and the Cu(II) concentration change was measured at various conditions.

#### 2.2.1. Time Course of Cu(II) Extraction with the Cryogel Containing LIX84-I

**Figure 6:**
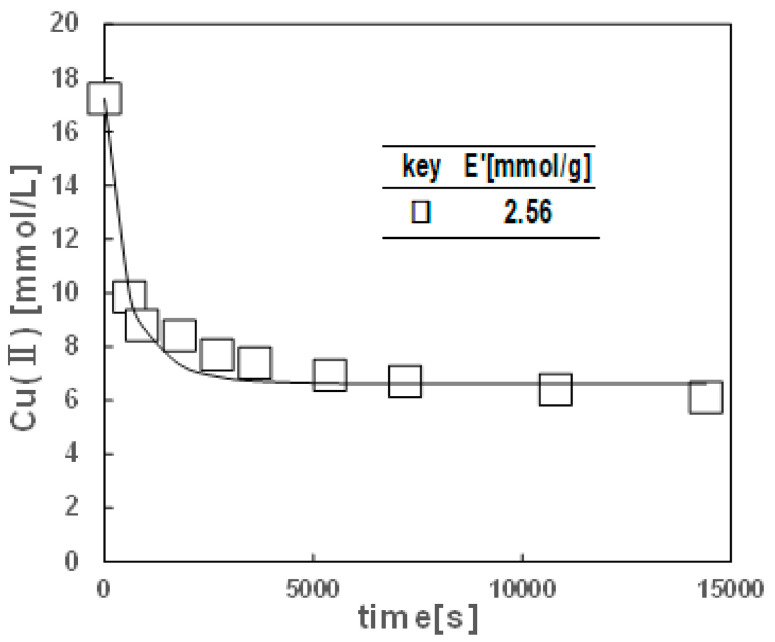
Time course of Cu(II) extraction with the hydrophobic cryogel containing LIX84-I at 2.56 mmol/g prepared at Trim 0.019 mol%, and −15 °C.

The time course of Cu(II) extraction with the hydrophobic cryogels containing LIX84-I is shown in Figure 6. The Cu(II) concentration in the aqueous solution decreased with time and reached a constant concentration which is equilibrium concentration. The decrease in Cu(II) concentration was very fast in the initial stage of the extraction, and changed to a slower decrease at around 1000 s and reached constant value after 6000 s. This rapid copper extraction in the cryogel in the initial stages is similar to that using LIX84-I impregnated polymer particles with inter-connected spherical pores [49]. In contrast, hydrophilic cross-linked gels encapsulating fine droplets of LIX84-I and commercial microporous particles impregnated with LIX84-I resulted in slower extraction, requiring several hours or more to become constant concentration of Cu(II) [49,53]. This hydrophobic cryogel containing LIX84-I, which has a similar structure to the LIX-84-I impregnated polymeric particles with cross-linked spherical pores [47,48], has a high specific surface area, and at the outermost surface of the cryogel, hydrophobic chains of the extractant are inserted into the hydrophobic polymer chain network that makes the cryogel walls and hydrophilic binding sites that interact with Cu(II) are exposed on the surface. This configuration is believed to contribute to an accelerated extraction rate.

The LIX84-I content of the cryogel was higher than that of LIX84-I-impregnated polymer particles (1 mmol/g), which is also considered to be advantageous for the high extraction rate of the cryogels. From the demonstration of extraction Cu(II), it seems potential that hydrophobic cryogel containing LIX84-I can be utilized for the removal of heavy metals from wastewater.

#### 2.2.2. Effect of Solvent Type and Trim Concentration Used for the Preparation on the Extraction Equilibria of Cu(II) with the Cryogels Containing LIX84-I

**Figure 7:**
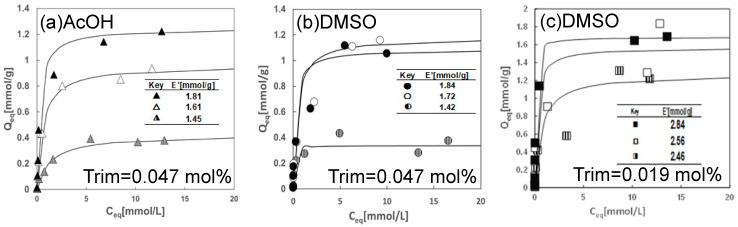
Effect of equilibrium concentration of Cu(II) on the extraction amount of Cu(II) in the cryogel, which were prepared with AcOH or DMSO at a Trim concentration 0.0047 mol% or 0.019 mol% in the reaction solution, and frozen at −15 °C.

The extraction equilibria of copper(II) from aqueous solutions were performed under various initial concentrations with hydrophobic cryogels containing LIX84-I prepared at a Trim concentration of 0.047 mol% or 0.019 mol% and at −15 °C in AcOH or DMSO. The extraction isotherms in AcOH or DMSO with various LIX84-I contents are shown in Figure 7. In all cases, the extracted amount of Cu(II) increased with an increase in the equilibrium Cu(II) concentration and reached constant values which increased with the LIX84-I content in the hydrophobic cryogel. Comparing the results using AcOH and DMSO at a Trim concentration of 0.047 mol%, the hydrophobic cryogel prepared in DMSO had a higher amount of the extracted Cu(II) at a lower concentration range of Cu(II). These higher extraction amounts for the DMSO-prepared cryogels may reflect the larger surface area of the cryogels prepared in DMSO.

In all conditions, the extraction behavior of Cu(II) into the hydrophobic cryogels conformed well to Langmuir-type adsorption as shown in Equation (1).

(1) $$Q_{eq}=\frac{Q_{max}KC_{eq}}{1+KC_{eq}},$$

where *Q_eq_*, *Q_max_*, *K*, and *C_eq_* are the molar equilibrium amount of Cu(II) extracted into the cryogels, maximum molar amount of Cu(II) in the cryogels, equilibrium constant, and equilibrium concentration of Cu(II) in the aqueous phase, respectively. The values of *Q_max_* and K obtained from the results in Figure 7 using Equation (2).

(2) $$\frac{1}{Q_{eq}}=\frac{1}{Q_{max}}+\left(\frac{1}{Q_{max}K}\right)\left(\frac{1}{C_{eq}}\right),$$

The solid lines in Figure 7 are the adsorption isotherm analyzed and calculated based on the Langmuir-type adsorption equation using the obtained values of *Q_max_* and *K*. The calculated adsorption isotherms are in good agreement with the experimental results as shown in Figure 7.

#### 2.2.3. Relationship between LIX84-I Content in the Hydrophobic Cryogel and Maximum Extracted Amount of Cu(II)

**Figure 8:**
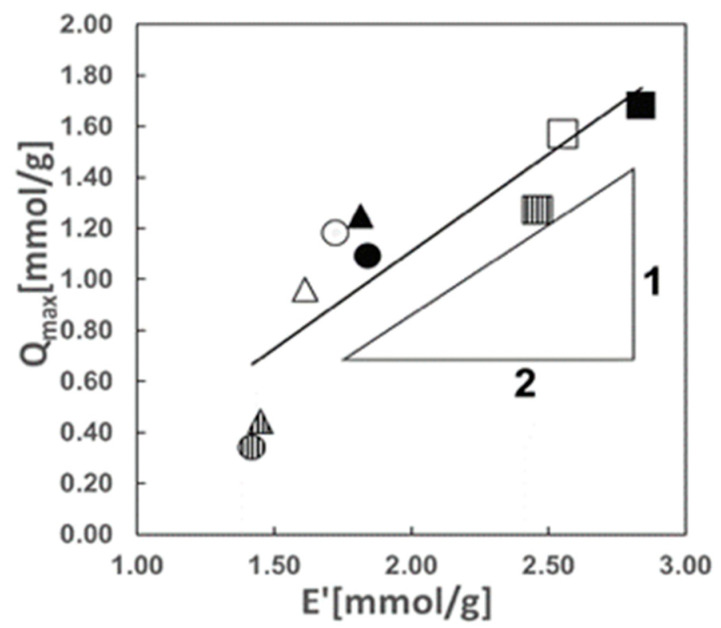
Relationship between the LIX84-I content in the hydrophobic cryogels used for the extraction, *E*′, and the maximum extracted amount of Cu(II) in the hydrophobic cryogels, *Q*_max_. The keys are the same as in Figure 7, which shows the hydrophobic cryogels used for Cu(II) extraction at different LIX84-I contents.

The relationship between the molar content of LIX84-I per gram of the polymeric particles, E*, and the maximum molar amount of Cu(II) extracted into the polymeric particles, *Q_max_*, gave a straight line with a slope of 1/2 when considering that the purity of LIX84-I is 52.4 wt% [48] as shown in Figure 8. It is assumed that two molecules of LIX84-I react with each Cu(II) ion. The stoichiometry of the complex reaction of LIX84-ICu(II) in the hydrophobic cryogels containing LIX84-I is shown below:

(3) $${\mathrm{Cu}}^{2+}+2\left(\mathrm{HR}\right)\to {\mathrm{CuR}}_{2}+2H^{+}\;\mathrm{HR}:\;\mathrm{LIX}84-I,$$

This equilibrium reaction between Cu(II) and LIX84-I in the cryogels is same as that in the polymer particles with inter-connected spherical pores impregnating LIX84-I [48] and that in PVA/Alg cross-linked gel microcapsules immobilized fine droplets of LIX84-I [53].

#### 2.2.4. Observation of the Hydrophobic Cryogels Containing LIX84-I in the Cu(II) Extraction Processes

**Figure 9:**
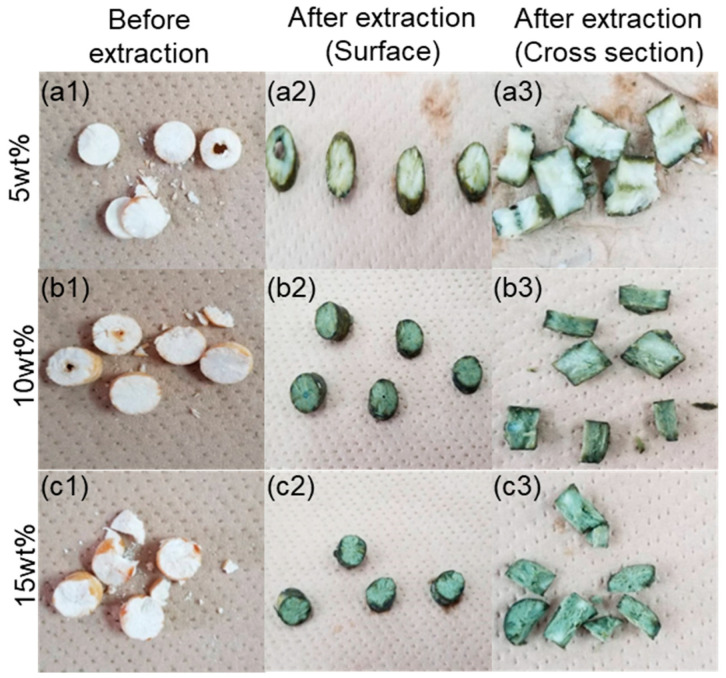
The photographs of cryogels were the before extraction (column #1, “#” shows the concentration of LIX84-I in the precursor solution; (**a1**–**a3**): 5; (**b1**–**b3**): 10; and (**c1**–**c3**): 15 wt%), and the after extraction on the surface (column #2) and the cross section (column #3) of the cryogels.

The photographs of the hydrophobic cryogels containing LIX84-I during the Cu(II) extraction process are shown in Figure 9. The cryogels prepared in DMSO at Trim concentration of 0.047 mol% and LIX84-I concentration of 5, 10, and 15 wt% in the precursor solution were used for the Cu(II) extraction, which is the same as in Figure 7b. Before extraction (left side line in Figure 9), the sides of the cylindrical hydrophobic cryogels were bright and light brown, while the center of the cylindrical cryogels were lighter in color. After Cu(II) extraction (midline for outer surface and right side for cross section in Figure 9), the surface of the cylindrical side surface was colored deep green, and the central part was lighter. This change to a deep green color indicates that the two molecules of LIX84-I form a complex with copper ions. The same color change was observed in the LIX84-I impregnated polymer particles [48]. The change in color intensity between the side surface and the center part in the cryogels would indicate that the concentration of LIX84-I is higher on the sides and lower in the center. This is thought to be due to phase separation and/or precipitation of LIX84-I in the non-frozen phase, which is mainly composed of LIX84-I and Trim, at the same time, because phase separation occurred due to crystallization of solvent from the wall side of the syringe at low temperature, resulting in a non-uniform concentration of LIX84-I in the cryogel.

Regardless of the heterogeneity of the concentration of LIX84-I in the hydrophobic cryogels, all LIX84-I molecules were used for extraction in the copper extraction equilibrium. This is indicated by the result that the maximum amount of LIX84-I extracted in the cryogels had a linear relationship with the LIX84-I contents in the cryogels having the slope of (1/2).

#### 2.2.5. Repeated Use of the Hydrophobic Cryogels Containing LIX84-I for the Cu(II) Extraction

After Cu(II) extraction with the hydrophobic cryogels containing LIX84-I prepared in DMSO and with a Trim concentration of 0.019 mol% and an LIX84-I concentration of 10 wt%, the precursor solution Cu(II) extracted in the cryogels could be back extracted using 0.5 M H_2_SO_4_ aqueous solution, and the back-extraction percentage of Cu(II) was over 100%. This means the extraction reaction of LIX84-I with Cu(II) at the pore surface of the hydrophobic cryogels proceeds reversibly.

**Figure 10:**
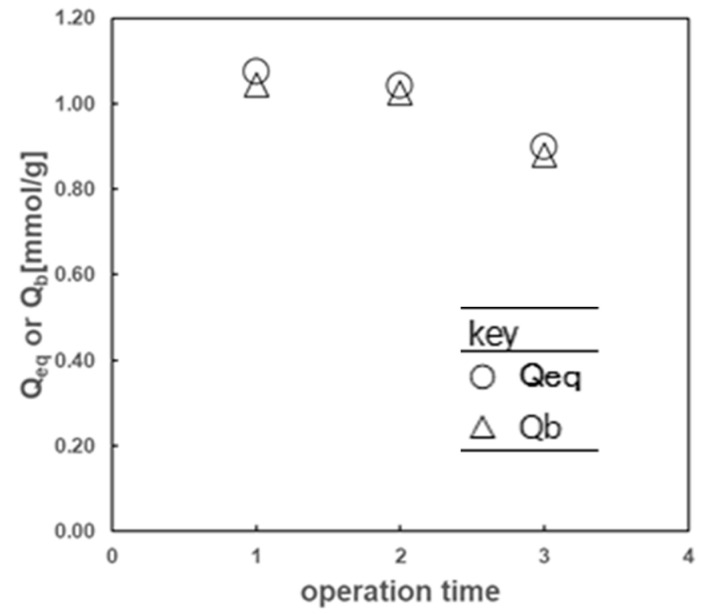
Effect of the repeated times of the extraction process on the extracted, Q_eq_, and back-extracted amounts, *Q_b_*, of Cu(II) with the hydrophobic cryogels containing LIX84-I.

Then, the hydrophobic cryogels containing LIX84-I were applied repeatedly for the following Cu(II) extraction and back-extraction process. The effect of repeated times on the extraction, *Q_eq_*, and back-extraction amounts, *Q_b_*, of Cu(II) are shown in Figure 10. There was little difference in the amount of extraction and the amount of back extraction at each number of repeated times. The amount of copper extracted did not decrease significantly even after repeated Cu(II) extraction process. This indicates that LIX84-I did not leak much from the cryogel during Cu(II) extraction. This would be because the use of hydrophobic cryogel as the immobilizing carrier allowed the hydrophobic extractant to be loaded and prevented the extractant from leaking into the aqueous phase during the repeated extraction process. Therefore, the hydrophobic cryogel is considered to be a good carrier of the extractant.

## 3. Conclusions

Supermacroporous hydrophobic cryogels were prepared by cryopolymerization at −15 °C using Trim as the monomer and DMSO or AcOH as the organic solvent. The pore volume and specific surface area of the hydrophobic cryogels were increased with a decrease in the freezing temperature and monomer concentration. By adding LIX84-I to the precursor solution of cryo-polymerization, hydrophobic cryogels with high LIX84-I content were obtained. The extraction of Cu(II) with the hydrophobic cryogels containing LIX84-I were as fast as that with the LIX84-I impregnated polymeric particles with cross-linked spherical pores in the initial stage of the extraction. The extraction amount of Cu(II) in the cryogels increased with the Cu(II) equilibrium concentration in the aqueous phase and reached constant at high Cu(II) concentration under all conditions. The maximum extraction amount increased with increasing LIX84-I contents in the cryogels. The extraction equilibrium was in good agreement with the experimental results, which were calculated according to the Langmuir-type adsorption isotherm. Dimethyl sulfoxide was the best solvent for the preparation of the hydrophobic cryogels containing LIX84-I. It was found that two molecules of LIX84-I react with one Cu(II) based on the relationship between the amount of LIX84-I contained in the hydrophobic cryogels and the maximum amount of Cu(II) extracted. These results suggest that the Trim-based hydrophobic cryogel is good carrier for highly hydrophobic extractants such as LIX84-I.

## 4. Materials and Methods

### 4.1. Materials

Trimethylolpropane trimethacrylate (Trim), 1,4-dioxane (DO, mp. 16.7 °C), acetic acid (AcOH, mp. 16.7 °C), dimethyl sulfoxide (DMSO, mp.), ethanol, and anhydrous copper sulfate (CuSO_4_) were purchased from Wako Pure Chemical Industry Ltd. (Japan). Benzoyl peroxide (BPO) and etlyl-4-dimethylaminobenzoate (EDMAB) were purchased from Tokyo Chemical Industry Ltd. (Tokyo, Japan). LIX84-I was used for extractant of Cu(Ⅱ). All materials were used as received without further treatment.

### 4.2. Preparation of Hydrophobic Cryogel

Trim and EDMAB were adopted as monomer and cross-linker to prepare hydrophobic cryogel. Table S1 shows the preparation condition of hydrophobic cryogel with different based organic solvent at different temperature for cryo-polymerization. Trim and LIX84-I were mixed with magnetic stirred and added to organic solvent. For the solvent, 1,4-dioxane (DO), acetic acid (AcOH), and dimethyl sulfoxide (DMSO) were adopted. Then the solution was purged with N_2_ gas for 5 min to remove dissolved oxygen to prevent the inhabitation of radical polymerization. Subsequently, cross-linker EDMAB and polymerization initiator were added to the solution and obtained polymer precursor. The obtained polymer precursor was quickly transferred to a 5 mL volume syringe (Outer diameter (*Φ* mm = 4.2)) and kept in cooling bath under frozen temperature of the solvent overnight. Here the cryo-polymerization was caused at 0, −5, −15 °C by setting the temperature of the cooling bath. The synthesized cryogel was thawed, washed, and dried under vacuum. Regarding containing the LIX84-I to hydrophobic cryogel, the LIX84-I was added in the range of 5–15 wt% during the process for the preparation of polymer precursor.

### 4.3. Appearance of Hydrophobic Cryogel

The hydrophobic cryogels prepared using different organic solvents; dioxane (DO), acetic acid (AcOH) and dimethyl sulfoxide (DMSO) were dried under vacuum after thawing for observation of their appearance and morphologies. The appearance of cryogels was recorded by using CCD camera.

### 4.4. Observation of Hydrophobic Cryogel

The microscopic morphologies of cryogels were observed by scanning electronic microscope (SEM, HITACHI, SU-5500 or Miniscope TM-1000) images. The accelerating voltage was set between 5 and 20 kV. The morphology of the cryogel prepared at temperature in the range of 0 to −15 °C (below mp. of solvent) during cryo-polymerization. As a pretreatment, the hydrophobic cryogel was coated with gold by using an ion coater.

### 4.5. Characterization of Porous Structure

A mercury intrusion porosimeter (MIP, AutoPore IV9500, Shimadzu, Japan) is a useful tool for characterization porous properties. MIP analysis is based on the intrusion mercury into pores by external pressure. The porous properties of hydrophobic cryogels preparation at different polymerization temperature were characterized from MIP analysis. In the characterization, porous properties of the cryogels were focused on total intrusion volume, total pore area, and mean pore size (*D_v_*), respectively.

### 4.6. Containing Amount of the Extractant

The amount of LIX84-I contained in hydrophobic cryogel was determined by using UV-Vis spectrometer (UV mini-1240, Shimadzu, Japan). The LIX84-I encapsulated cryogel was kept in 20 mL ethanol for 8 h at room temperature and was eluted LIX84-I in the ethanol solution. The supernatant was obtained from the cryogel by filtration and the eluted solution. The concentration of LIX84-I (*C_LIX84-I_*) in the solution was calculated from UV absorbance at 270 nm. The encapsulated amount of LIX84-I (*E′*) to the hydrophobic cryogel were calculated according to Equation (4).

(4) $$E^{\prime }=\frac{C_{LIX84-I}V_{EtOH}}{W_{cryogel}}$$

where *V_EtOH_* and *W_cryogel_* are represented volume of ethanol and weight of cryogel, respectively.

### 4.7. Extraction of Cu(Ⅱ) with the Hydrophobic Cryogel Containing LIX84-I

Cylindrical cryogels were cut transversely into discs approximately 5 mm thick, weighed, and used for copper extraction. The extraction of Cu(II) ions can be characterized by batch extraction experiments. The extraction of Cu(Ⅱ) by using hydrophobic cryogel containing LIX84-I was performed in 30 mL of CuSO_4_/NH_3_ solution, where pH = 4.5. The disc type cryogels were incubated in CuSO_4_/NH_3_ solution at 30 °C for 24 h. The Cu(II) concentrations in the filtrate, feed, and back-extracted solutions were measured by ICP-AES (ICPS-8100 Shimadzu). The amounts of Cu(II) extracted into the cryogels and back-extracted from the cryogels were calculated from the mass balances between the initial and equilibrium amounts of Cu(II) in the aqueous solutions and in the cryogels, respectively [14]. Following incubation, the visual characteristics of the hydrophobic cryogels containing LIX84-I were documented at various stages, including before extraction and after extraction, encompassing both surface and cross-sectional views.

## Figures and Tables

**Table 1:** Porous analysis of hydrophobic cryogels at different polymerization temperatures.

Entry	Solvent	*T* [°C]	Total Pore Volume [mL/g]	Total Pore Area [m^2^/g]	*D_v_* [μm]
8		0	n.d. *^a^*	n.d. *^a^*	n.d. *^a^*
5	AcOH	−5	0.1599	0.064	10.00
2		−15	1.7795	0.306	23.23
9		0	1.8032	0.338	9.99
6	DMSO	−5	1.4380	0.435	13.23
3		−15	1.8032	0.748	20.35

*^a^* and *D_v_* represented as no data and mean pore size, respectively. Entry No. are the same as those in Table 1.

## Data Availability

The data presented in this study are openly available in article.

## Supplementary Materials

The following supporting information can be downloaded at: https://www.mdpi.com/article/10.3390/gels10010009/s1, Table S1: Preparation condition of hydrophobic cryogels.
