# Supplementary material for: Preparation of Hydrophobic Cryogel Containing Hydroxyoxime Extractant and Its Extraction Properties of Cu(Ⅱ)
Source: Gels. 2023 Dec 21;10(1):9. doi: 10.3390/gels10010009 (PMC10815328; doi:10.3390/gels10010009)
Supplement: Supplementary file 1 [file gels-10-00009-s001.zip › gels-2764338-supplementary.pdf]

Supporting Information

Preparation of Hydrophobic Cryogel Containing Hydroxyoxime Extractant and Its Extraction Properties of Cu(Ⅱ)

Hayato Takase [1], Naoto Goya [2], Shiro Kiyoyama [3], Koichiro Shiomori [4] and Hideki Matsune [4,*]

[1] Department of Chemical Engineering, Graduate School of Science and Engineering, Kagoshima University, 1-21-40 Korimoto, Kagoshima 890-0065, Japan
[2] Department of Applied Chemistry, Graduate School of Engineering, University of Miyazaki, 1-1 Gakuenkibanadai-nishi, Miyazaki 899-2192, Japan
[3] Department of Chemical Science and Engineering, National Institute of Technology, Miyakonojo College, 473-1 Yoshi-cho, Miyakonojo-shi, Miyazaki 885-8567, Japan
[4] Department of Applied Chemistry, University of Miyazaki, 1-1 Gakuenkibanadai-nishi, Miyazaki 899-2192, Japan
* Correspondence: matsune@cc.miyazaki-u.ac.jp; Tel.: +81-985-58-7306

Keywords: Hydrophobic cryogel; Cryo-polymerization; Frozen temperature of organic solvent; Supermacroporous structure; Extraction properties of Cu(Ⅱ).

Table S1. Preparation condition of hydrophobic cryogel

Entry	DO [g]	AcOH [g]	DMSO [g]	Trim [g]	BPO [g]	EDMAB [g]	T [ºC][a]
1	25.825	-	-	4.907	0.702	0.558	-15
2	-	26.25	-	7.445	1.066	0.846	-15
3	-	-	27.51	5.956	0.853	0.677	-15
4	25.825	-	-	4.907	0.702	0.558	-5
5	-	26.25	-	7.445	1.066	0.846	-5
6	-	-	27.51	5.956	0.853	0.677	-5
7	25.825	-	-	4.907	0.702	0.558	0
8	-	26.25	-	7.445	1.066	0.846	0
9	-	-	27.51	5.956	0.853	0.677	0
10	-	26.25	-	14.89	1.066	0.845	-15
11	-	-	27.51	11.91	0.853	0.677	-15
12	-	26.25	-	2.863	1.066	0.846	-15
13	-	-	27.51	2.978	0.853	0.677	-15
14			27.51	1.489	0.853	0.677	-15

[a]Means the temperature of cryo-polymerization.
